# Treatment of chronic non-healing ulcers using autologous platelet rich plasma: a case series

**DOI:** 10.1186/s12929-017-0324-1

**Published:** 2017-02-27

**Authors:** Manish Suthar, Saniya Gupta, Suhail Bukhari, Venkatesh Ponemone

**Affiliations:** 1 TotipotentRX Center for Cellular Medicine (Cesca Therapeutics Inc., USA), Fortis Memorial Research Institute, Gurgaon-122002, Delhi, (NCR) India; 2 Fortis Escorts Heart Institute and Research Center, New Delhi, India

**Keywords:** Autologous platelet rich plasma, Non-healing ulcers, Point-of-care, Cellular therapy, Cytokines, Growth factors

## Abstract

**Background:**

Non-healing ulcers are a major health problem worldwide and have great impact at personal, professional and social levels, with high cost in terms of human and material resources. Recalcitrant non-healing ulcers are inevitable and detrimental to the lower limb and are a major cause of non-traumatic lower limb amputations. Application of autologous Platelet Rich Plasma (PRP) has been a major breakthrough for the treatment of non-healing and diabetic foot ulcers, as it is an easy and cost-effective method, and provides the necessary growth factors that enhance tissue healing. PRP is a conglomeration of thrombocytes, cytokines and various growth factors which are secreted by α-granules of platelets that augment the rate of natural healing process with decrease in time. The purpose of this case series was to evaluate the safety and efficacy of autologous platelet rich plasma for the treatment of chronic non-healing ulcers on the lower extremity.

**Methods:**

Autologous PRP was prepared from whole blood utilizing a rapid, intraoperative point-of-care system that works on the principle of density gradient centrifugation. Twenty Four (24) patients with non-healing ulcers of different etiologies, who met the inclusion criteria, were treated with single dose of subcutaneous PRP injections along with topical application of PRP gel under compassionate use.

**Results:**

The mean age of the treated patients was 62.5 ± 13.53 years and they were followed-up for a period of 24 weeks. All the patients showed signs of wound healing with reduction in wound size, and the mean time duration to ulcer healing was 8.2 weeks. Also, an average five fold increase in the platelet concentrate was observed in the final PRP product obtained using the rapid point-of-care device, and the average platelet dose administered to the patients was 70.10 × 10^8^.

**Conclusion:**

This case series has demonstrated the potential safety and efficacy of autologous platelet rich plasma for the treatment of chronic non-healing ulcers.

**Trial registration:**

NCT03026855, Registered 4 January 2017 ‘Retrospectively’

## Background

Chronic ulcers or non-healing ulcers are defined as spontaneous or traumatic lesions, typically in lower extremities that are unresponsive to initial therapy or that persist despite appropriate care and do not proceed towards healing in a defined time period with an underlying etiology that may be related to systemic disease or local disorders [1, 2]. There are many types of non-healing ulcers that may include venous, arterial, diabetic, pressure and traumatic ulcers. The normal wound healing process is dynamic and complex having three phases: inflammation, tissue formation and tissue remodeling. However, if the normal healing process is interrupted, an ulcer can become chronic in nature due to lack of growth factors and cytokines which delay the healing process [3]. Lower extremity ulcers comprise a substantial proportion of chronic ulcers, especially those attributed to venous disease, diabetes, or arterial disease [2]. Chronic non-healing ulcer is a major health problem and is estimated to affect approximately 2–6 million people in the United States alone [2, 4], while its prevalence in the world ranges from 1.9 to 13.1% [5, 6]. The incidence of chronic ulcers is expected to increase as the population ages and due to increased risk factors for atherosclerotic occlusion such as smoking, obesity and diabetes. It is estimated that almost 10% of the population would develop a chronic wound in the course of a lifetime, with wound related mortality rate of 2.5% [6]. These types of ulcers not only affect the quality of life and productivity of the patient but also become a substantial financial burden for the patient and the healthcare system [7].

“More than 85% of lower limb amputations are preceded by foot or ankle ulcers and diabetes is one of the major causes of non-traumatic amputations across the world” [8]. Approximately 15–25% of individuals with diabetes develop a foot ulcer, of which an estimated 12% require lower extremity amputation [2]. Individuals with diabetic foot ulcers are susceptible to infection and the healing process is complicated by diabetic neuropathy leading to chronic non-healing ulcers. Majority of the chronic lower extremity ulcers are accounted by venous disease as venous hypertension results in damage to vessel walls and ultimately lead to skin breakdown [2]. The prevalence of venous non-healing ulcers varies between 1 and 2% in the general population that accounts for almost 75–80% of all vascular ulcers [1, 9, 10].

The goal of ulcer treatment is to obtain wound closure as expeditiously as possible. Conventional treatment for non-healing ulcers includes wound cleansing, necrotic tissue debridement, prevention, diagnosis, and, if necessary, treatment of infection, mechanical off-loading, management of blood glucose levels and local ulcer care with dressing application [2, 11, 12]. However, there are certain risk factors that commonly affect and contribute to poor wound healing, these include: 1) Local causes, such as presence of debris or necrotic tissue, infection in the ulcer, tissue hypoxia, and repeated trauma; 2) Systemic diseases, such as diabetes mellitus, immunodeficiency, or malnutrition; and 3) Medications, such as corticosteroids [3].

The standard available treatment modalities for non-healing ulcers address these issues and provide optimal local ulcer therapy with debridement of necrotic tissue and provision of a moist wound healing environment, pressure relief in the wound area, infection management using antibiotics, antiseptics and topical antibacterial agents, ischemia management, and medical management of comorbidities. A wide variety of advanced treatment for non-healing ulcer include hyperbaric oxygen therapy, skin grafting, VAC (vacuum assisted closure) and surgical management like angioplasty and reconstructive surgery as needed [3, 13, 14].

Despite treatment, many chronic ulcers fail to heal or persist for months/years and/or recur after healing, requiring additional advanced wound care therapies for adequate healing [3]. Cellular therapy for the treatment of non-healing ulcer has been a major breakthrough in the arena of vascular therapies. The use of patient’s own body cells for wound/ulcer treatment relies upon the components present in the blood and platelet concentrate, which contains various cytokines and growth factors. These modular treatment options are safe and effective and have no side effects. Over the last two decades, emerging cellular therapies such as platelet-rich plasma (PRP) therapy has gathered considerable attention for its potential use in the field of regenerative medicine as a therapeutic agent in a range of chronic conditions and can have an adjunctive role in a standardized, quality treatment plan [13, 15]. Autologous PRP is a platelet suspension in plasma derived from whole blood that is increasingly being used in clinical practice for the treatment of chronic ulcers. The concentration of platelets in PRP is 2–6 folds higher than that of whole blood [1, 16]. The curative properties of PRP rely on the fact that platelets are a physiological reservoir of a variety of growth factors, with healing function which have an active role in tissue regeneration [15].

PRP is increasingly being used as a new alternative approach in various fields of medicine (i.e. dentistry, traumatology, cosmetic surgery, ophthalmology, and dermatology). Platelets contain proteins, known as growth factors that trigger biological effects including directed cell migration (i.e. chemotaxis), angiogenesis, cell proliferation and differentiation, which are key elements in the process of tissue repair and regeneration. Several studies have also been published on the role of platelet rich plasma for the treatment of non-healing ulcers with positive response [17].

PRP is most often mixed with thrombin before application in order to generate a fibrin gel, and a platelet-growth-factors-rich exudate [18]. Thrombin activated platelets release numerous growth factors from their $\alpha$-granules [19] that can modulate cell proliferation and differentiation and accelerate soft tissue repair in vivo [20].

A recent meta-analysis of the use of PRP therapy in cutaneous wounds showed that compared to control wound care, PRP facilitated wound healing and the ulcers improved significantly in small hard-to-heal acute and chronic wounds [21, 22]. In addition, platelets exert antimicrobial activity against some bacteria of the skin, and clinical data shows that the presence of infection is reduced in PRP-treated wounds [22]. Therefore, PRP therapy has several advantages that can provide a practical and effective treatment approach for small hard-to-heal ulcers [23].

Autologous Platelet Rich Plasma treatment for Non-healing ulcers has been a breakthrough in the stimulation and acceleration of soft-tissue healing. PRP therapy helps create a biological environment internally that is most conducive for restoration of tissue homeostasis by providing numerous signalling cytokines and growth factors that are important in tissue repair by diverse mechanisms including the regulation of inflammation, angiogenesis, and synthesis and remodelling of new tissue [15]. The advantages and merits of PRP are apparent since it is easy, cost-effective and much more lasting compared to other standard treatments and being autologous in nature, it is free from communicable pathogens, making it a safe treatment modality with good clinical results [10, 21].

The purpose of the present case series was to evaluate the safety and efficacy of autologous PRP in treating non-healing ulcer on lower extremity using a rapid, intra-operative point-of-care technology at the patients’ bed side.

## Methods

### Patient selection criteria

In this case series 24 patients between the age group 18–85 years, with chronic or non-healing ulcers of various aetiologies (such as pressure ulcers, venous ulcers, arterial ulcers or diabetic foot ulcers) who were treated with autologous PRP under compassionate use were included. Patients with an ulcer of at least 4-weeks’ duration were eligible if they met additional inclusion/exclusion criteria: Index foot ulcer located on the plantar, medial, or lateral aspect of the foot (including all toe surfaces); and wound area (length x width) measurement between 0.5 and 10 cm^2^, inclusive. The index ulcers had to be clinically non-infected (infection was diagnosed through clinical signs and symptoms rather than culture results) and full-thickness without exposure of bone, muscle, ligaments, or tendons. Smokers and individuals with systemic disease or history of anticoagulant, immunosuppressive, or antibiotic therapy in the last 3 months were excluded. Additionally, pregnant women, patients with severe cardiovascular disorder and patients with a bleeding disorder and uncontrolled sugar levels were excluded. Patient history, physical examination, and data of routine investigations were obtained.

Those patients who met the inclusion criteria were explained the entire treatment and follow-up procedure by the study investigator, and only after obtaining voluntary informed consent from the patients for the treatment procedure, they were treated with PRP and their follow-up data collected. The procedure was conducted in accordance to the Declaration of Helsinki, and all care was taken to maintain patient safety and confidentiality. The study was approved by ‘The Independent Ethics Committee (TIEC)’ (TIEC20123919) of the participating institution and registered with clinicaltrials.gov (Identifier: NCT03026855). All the included patients received single dose of autologous PRP injections that was processed using a 510(k) approved device and PRP gel application over the wound/ulcer on the day of treatment procedure.

### Preparation of Platelet Rich Plasma (PRP)

PRP was prepared using an advanced rapid point-of-care technology, the Res-Q™ 60 PRP system (Thermogenesis Corp., USA) at the patient’s bed side. This point-of-care system is an automated, closed, sterile system that processes and concentrates whole blood by density gradient centrifugation in less than 15 min. The treatment procedure is non-invasive using minimally manipulated autologous platelet concentrate, thereby decreasing the chances of infection and cross contamination while processing and administration of the cellular therapy. Briefly, depending upon the size of ulcer or wound, and under aseptic conditions, 40 to 60 ml of peripheral blood was drawn in syringes containing anticoagulant (Acid Citrate Dextrose – ACD-A) in the ratio of 3:17 (anticoagulant: whole blood) from patient’s anti-cubital vein using a 21-gauge needle. Blood and anticoagulant were thoroughly mixed before transferring to the processing device, to prevent formation of blood clots, which in turn facilitates higher cell recovery. At this time, 1 mL aliquot of pooled blood was segregated and later analyzed for pre-processed platelet counts and sterility. The aspirated whole blood was then processed using the Res-Q™ 60 PRP processing device at the patient’s bedside. The device works by separating peripheral blood into three distinct layers; Erythrocytes settle at the substratum, above that the plasma layer containing rich concentrate of platelets (PRP) and platelet poor plasma (PPP) as the top layer. After centrifugation, 7 mL of PRP was harvested from the processing device using aseptic technique, of which 1 mL aliquot was separated for post-processed platelet counts and sterility analysis. The remaining 6 mL was transferred to the sterile field for subcutaneous injections and activation of platelets for gel formation.

### Preparation of activator solution

Activator solution was prepared by combining human thrombin (500 IU/ml) (Baxter AG, Austria) with 1% Calcium Chloride (CaCl_2_) in the ratio of 1:9, respectively_._ The combination of Thrombin and Calcium Chloride activates platelets in just fraction of seconds resulting in fibrin matrix formation. Depending upon the wound area, 2-3 mL of activator solution was prepared in the sterile field and kept aside for application along with PRP. Calcium chloride nullifies the effect of the acid citrate anticoagulant used, and thrombin helps in activating fibrinogen, which is converted to fibrin and cross-linked [24].

### Treatment procedure

The non-healing ulcers were first debrided to remove any necrotic and infected tissues and the wound area was cleaned thoroughly with betadine solution. Based on the wound size and area, 2–3 mL of PRP was aliquoted from the 6 mL prepared PRP solution for gel formation, while the remaining 3–4 mL was injected subcutaneously inside and around the periphery of the wound/ulcer. Autologous platelet gel was obtained by spraying simultaneously equal volumes of PRP and activator solution (thrombin with calcium chloride) using a ratio applicator duploject system (dual port injection syringe that permits the simultaneous injection of PRP and activator solution), topically over the ulcer or wound. Within 5 to 10 s, a platelet gel was formed on the wound, following which a non-absorbent dressing was used to cover the wound/ulcer area (non-absorbent sterile transparent sheet, Tegaderm™, 3 M Medical Inc.). The dressing was changed on day 3 post-treatment; the wound was irrigated with normal saline and assessed for the presence of any form of infection. Following which the dressing was frequently changed once a week and the patients were followed up for a period of 24 weeks post-treatment. Care and management efforts provided at each treatment visit included cleansing and assessing the wound and obtaining vital signs and an interim wound history, including information regarding adverse events, concomitant medications, nutrition and weight-bearing status, and other aspects of care since the last visit. Wounds were photographed before treatment and at each follow-up visit after treatment using a digital camera.

### Endpoints

Our primary objective was to assess the efficacy of PRP in wound/ulcer healing by evaluating the percentage reduction in wound/ulcer size over the 24 weeks follow-up period by visual inspection. The secondary objectives included safety and feasibility of autologous PRP injections, time to wound/ulcer healing, improvement in pain or discomfort, and quality of life.

## Results

**Fig. 1:**
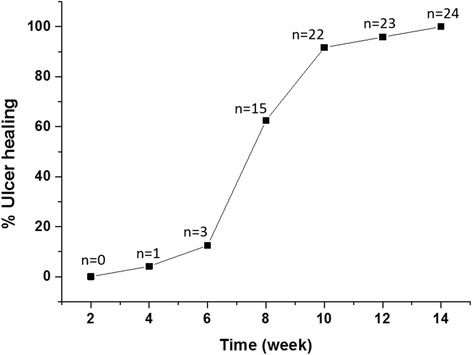
Distribution of cumulative ulcer healing time following PRP treatment

Twenty-four patients, each having one wound/ulcer of varying etiology were included and treated with single dose of PRP injections around the wound periphery and topical administration of autologous platelet gel. Among the included patients, 16 (66.6%) were males and 8 (33.33%) were females with a mean age of 62.5 ± 13.53 years. Out of 24 patients, 16 (66.6%) were in 61–80 years age group, 7 (29.17%) were in 41–60 years age group, and only 1 (4.17%) patient was <40 years. Additionally, among the ulcers treated, there were 10 (41.67%) venous ulcers, 9 (37.5%) diabetic ulcers, 3 (12.5%) arterial ulcers and 2 (8.33%) pressure ulcers. The duration of the non-healing ulcers presented by the patients’ pre-treatment ranged from 9 to 24 weeks with a mean duration of 16 weeks (Table 1). Wound/ulcer healing was observed as early as 4 weeks post-PRP treatment and the mean healing time was found to be almost 8.2 weeks ± 1.9 (Fig. 1). Four weeks after the application of PRP gel, small islands of granulating tissue appeared over the wound, and a significant reduction in wound size and increase in tissue mass was observed as a sign of healing and improvement.

**Table 1:** Duration the wound/ulcers persisted before PRP treatment

Duration of Ulcer (weeks)	No. of Patients (n)	Percentage (%)
9–12	4	16.67
13–16	9	37.50
17–20	8	33.33
21–24	2	08.33

All the patients showed healing of the wound/ulcer where more than 90% reduction in wound size was observed in 17 (70.83%) patients, followed by 80–90% reduction in wound size in 3 (12.5%) patients over the 24 weeks follow-up post-PRP application (Table 2). Overall, significant reduction in wound size was observed in all the treated patients.

**Table 2:** Percentage Improvement in Wound/Ulcer Healing after PRP treatment

Reduction in ulcer size at the end of follow-up	No. of Wound/ulcer	Percentage (%)
≤60%	01	4.17
61–70%	01	4.17
71–80%	02	8.33
81–90%	03	12.50
> 90%	17	70.83

**Fig. 2:**
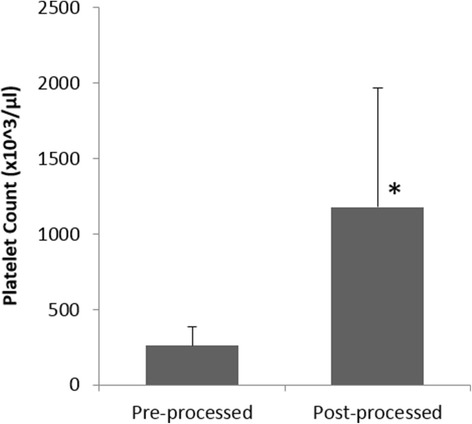
Distribution of Platelet counts at pre- and post- processing (*p < 0.05)

**Fig. 3:**
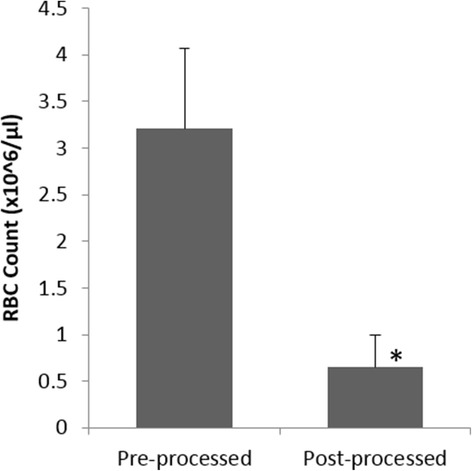
Distribution of Reduction in RBC content in Pre- and Post-processed PRP (*p < 0.05)

**Fig. 4:**
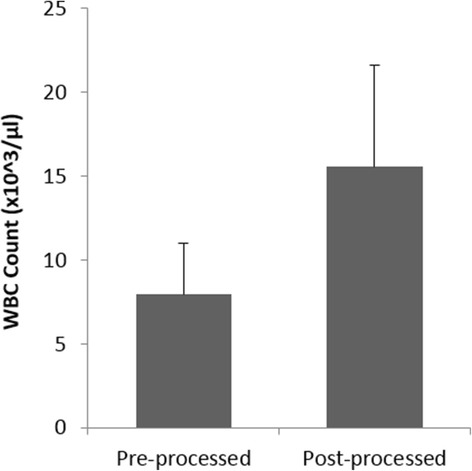
Distribution of WBC counts in pre- and post- processed PRP

PRP processing and administration was accomplished at the patient’s bedside in a single sitting within 30 min using the Res-Q™ 60 PRP system. Patients tolerated the procedure well, and there was no bleeding, infection, or procedure related complication, including local injection site swelling in all the subjects on the day of treatment after PRP injection and autologous PRP gel application. The mean (±Standard Deviation) platelet counts increased from 261.91 × 10^3^ (±125.31)/μL to 1177.35 × 10^3^ (±787.95)/μL in the post-processed sample (Fig. 2). Almost five fold increase in the platelet counts were observed in the final PRP product, which was statistically significant (*p* < 0.05). Furthermore, there was a statistically significant (*p* < 0.05) reduction in the Red Blood Cell (RBC) counts in the post processed sample as compared to whole blood, where the counts reduced by almost 80% and the mean RBC content in the final PRP product was 0.65 × 10^6^ (±0.34)/μL (Fig. 3). The mean White Blood Cell (WBC) Count of all the patients was 15.6 × 10^3^ (±5.93)/μL in the concentrated PRP, which was considerably higher than the mean WBC count in whole blood (7.94 × 10^3^ (±3.04)/μL) (Fig. 4), and the mean platelet dose administered to the patient’s was 70.10 × 10^8^.

**Fig. 5:**
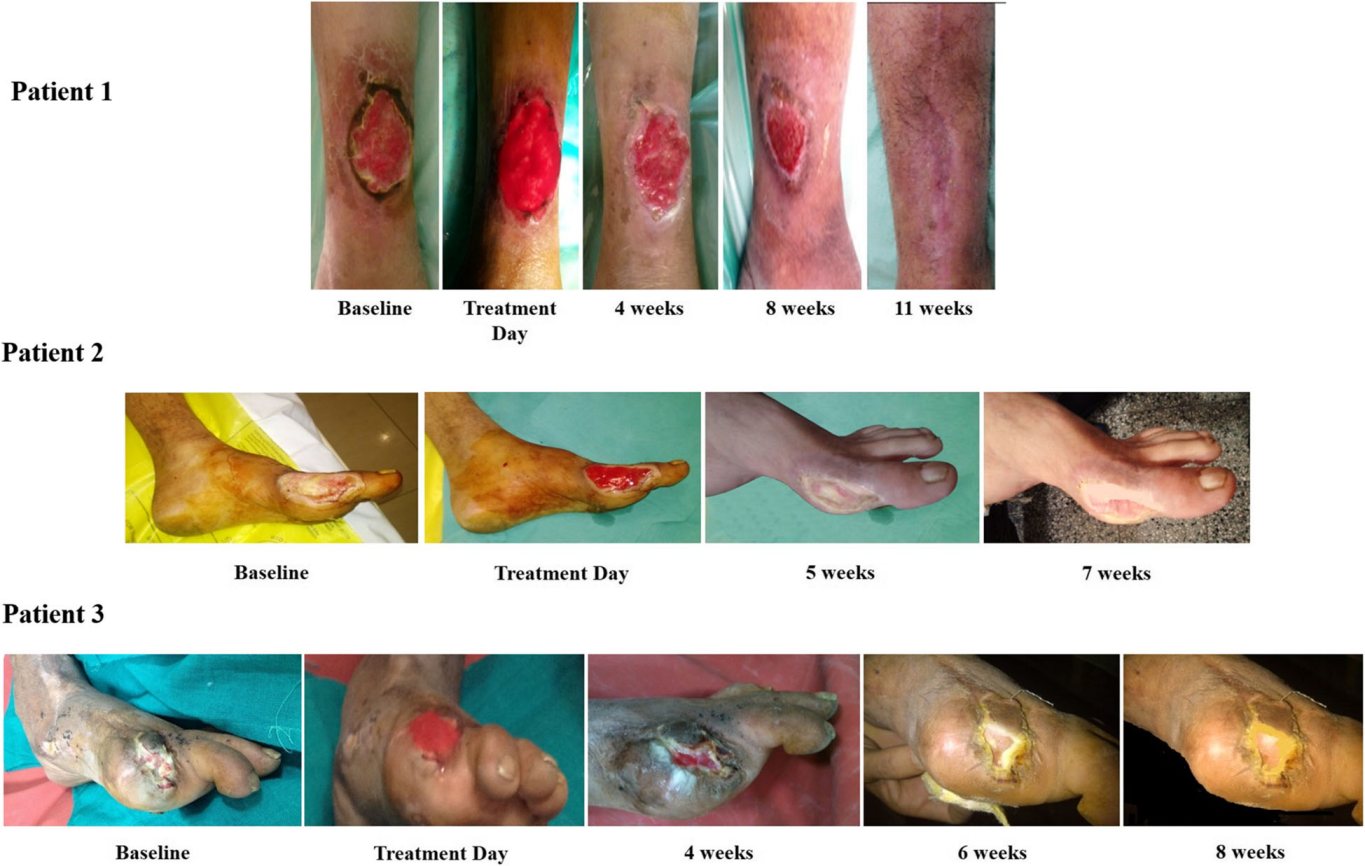
Patient follow-up images depicting the treatment efficacy of PRP during follow-up period

Figure 5 shows the patient follow-up pictures, pre- and post- PRP treatment, which clearly depicts the reduction in wound/ulcer size with time following PRP administration. Reduction in pain and serous discharge from wounds was noted within 1 week post-treatment and this could be due to the anti-inflammatory property of PRP, which contains leukocytes. Also, no adverse effects were reported on the day of treatment and during the patient’s follow-up period, deeming this as a safe and effective treatment method for chronic non-healing ulcers. Furthermore, the overall quality of life of the treated patients improved tremendously post- PRP therapy.

## Discussion

Leg ulcers are classified as acute or chronic according to the duration they have persisted; However, there is no specific length of time to define chronicity [7]. Chronic wounds/ulcers come with significant cost and morbidity for the patients and society as a whole. These non-healing ulcers of lower extremity develop as a result of peripheral neuropathy, ischemia, or trauma [25] and are often difficult to treat. The main goal of any treatment modality is to obtain wound closure expeditiously. The conventional treatment includes adequate debridement, control of infection, re-vascularization of ischemic tissue, and avoidance of undue pressure on the wound. Skin grafting has shown some efficacy, however they are not capable of providing the necessary growth factors to modulate the healing process and are expensive [10, 23].

In 1986, Knighton et al. showed that the use of autologous platelet factors accelerated epithelialization of granulation tissue leading to complete repair of chronic non-healing ulcers. This was the first clinical study that demonstrated the promising role of locally acting factors derived from autologous blood in promoting healing of chronic cutaneous ulcers [26]. Platelets contain a large number of growth factors and cytokines that play key roles in inflammation and tissue repair, by contributing towards haemostasis at sites of vascular injury. These characteristics of platelets have led to the idea of using platelet rich plasma as a therapeutic tool to promote wound healing, particularly in patients whose tissue repair is significantly impaired or delayed [27, 28].

PRP is a rich concentrate of platelets, cytokines and growth factors dispersed in a very small amount of plasma which can be prepared from a sample of centrifuged autologous blood. The α-granules of platelet rich plasma contain various growth factors primarily Platelet Derived Growth Factor (PDGF), Vascular Endothelial Growth Factor (VEGF), Transforming Growth Factor-β (TGF-β), Insulin-like Growth Factor (IGF) and Fibroblast Growth Factor (FGF) to name a few that locally attract progenitor cells to stimulate proliferative and differentiation activities and improve wound healing via autocrine and paracrine mechanisms [16, 27, 28]. Platelets initiate the wound healing process through release of locally active growth factors [21, 29–31] that attract undifferentiated cells to the site of injury and promote their cell division. PRP may also limit inflammation by suppressing cytokine release at the site of injury, and further improve the regeneration process by promoting capillary angiogenesis and re-epithelialization because of the presence of large amount of leukocytes [10, 32]. Our results also reported the presence of 15.6 × 10^3^ (±5.93)/μL mean leukocytes in the PRP concentrate which was almost two fold higher than the leukocyte count in the pre-processed whole blood sample. Growth factors from platelets are released only upon activation, wherein approximately 70% of the stored growth factors in the platelet are released within the first 10 min. Complete release of platelet growth factors is accomplished within 1 h of activation [16]. Therefore, it is recommended to activate platelets just before applying PRP on to the ulcer area.

The growth factors released from PRP are important in modulating mesenchymal cell recruitment, proliferation and extracellular matrix synthesis during the healing process [33]. PDGF stimulates chemotaxis, proliferation and new gene expression in monocytes, macrophages and fibroblasts in vitro, and these cell types are considered essential for tissue repair. Transforming growth factor-β stimulates cell proliferation, protein synthesis and collagen synthesis. It also inhibits growth of many epithelial tumour cells and fibroblastic cell lines. Platelet derived angiogenesis factor is a polypeptide capable of stimulating new capillary growth by inducing migration of endothelial cells. Platelet derived epithelial cell growth factor is partially responsible for the initial influx of neutrophils into the wound space; it is also a mitogen for many cells, including epithelial cells and fibroblasts. More recently, it was suggested that this was the mechanism by which platelet factors influence the process of angiogenesis and revascularisation, thus promoting granulation tissue formation [7]. In addition to growth factors, leucocytes also help in wound healing as they help in preventing infections.

Activated platelets, apart from growth factors, also release large amounts of elements that contribute to primary homeostasis, like fibrinogen, serotonin, fibronectin, factor V, factor VIII (Von Willebrand factor) and calcium (factor IV). These result in formation of platelet aggregates (clots), causing platelet stabilization by cross-linked fibrin and sticky glycoproteins. The formed fibrin matrix promotes cell permeation with monocytes, fibroblasts and other progenitor cells that play an important role in ulcer healing [16]. A major advantage of PRP over the use of single recombinant human Growth Factor delivery is the release of multiple growth factors and differentiation of factors upon platelet activation [10, 34].

In our case series, 24 patients with one wound/ulcer per patient were treated with a single dose of a combination of autologous PRP gel and subcutaneous injections of PRP in and around the wound periphery. All the patients showed healing of the wound with reduction in wound size, and the mean time to healing of the ulcers was 8.2 ± 1.9 weeks. Reduction in pain was observed in all the patients post-treatment and also, the quality of life of the patients significantly improved. The results demonstrated the safety and efficacy of autologous PRP in treating chronic non-healing ulcers. PRP was prepared using a rapid point-of-care device, the Res-Q™ 60 PRP system, an autologous platelet separator instrument designed to be used at the patient’s bedside for the safe and rapid preparation of platelet rich plasma from peripheral blood. The device concentrated platelets three to five (3–5) fold higher as compared to baseline values in whole peripheral blood with minimal manipulation. Additionally, the point-of-care approach avoids the need for a second procedure (at a different time point) to harvest and implant cells making it more cost-effective (≈USD280 per procedure) and this type of therapy also circumvents many of the limitations of exogenous cell therapy by avoiding in vitro cell manipulation, costly cell expansion, and the need for Good Manufacturing Practice (GMP) facility.

A study conducted by Frykberg et al., on 49 patients with 65 non-healing ulcers showed that 63 of 65 ulcers responded with a reduction in area, volume and undermining of the ulcers in a mean duration of 2.8 weeks with 3.2 treatments [35]. Another study by Kakudo et al., treated five cases of intractable skin ulcer with autologous PRP, among which three ulcers healed completely within 4 weeks and epithelialiazation of wound occurred within 6.6 weeks on average [28]. A prospective, randomized, controlled, blinded multicenter study conducted by Driver et al., initially included 72 patients with diabetic foot ulcers who were treated with autologous platelet-rich plasma gel or control (saline gel). However, 32 patients were excluded from the final protocol because of protocol violations and failure to complete treatment. Their study results showed that significantly more wounds healed in patients treated with platelet-rich plasma gel (13 out of 16 or 81.3%) than patients treated with control gel (eight out of 19 or 42.1%). However, the study had several limitations including small sample size, protocol violations occurring during the study period, and high rate of patient dropouts [13]. Furthermore, Steenvoorde et al., conducted a study on 12 patients with 13 wounds, showing that seven of 13 wounds required more than one application, with a mean number of 2.2 applications and a mean treatment period of 4.2 weeks [36]. The results from our case series were concurrent with previously published studies in terms of healing time, even though in our study only a single dose of PRP was administered.

Several studies have been conducted on the use of PRP for the treatment of non-healing ulcers and the results have been promising, however, currently, there is a paucity of critical scientific data regarding the beneficial effects of PRP in clinical procedures. PRP is an autologous preparation, making it a safe treatment modality as compared to allogenic preparations and is free from concerns over transmissible diseases [21, 37]. Moreover, PRP requires no special considerations regarding antibody formation, thereby, effectively preventing the risk of graft vs. host disease and leading to better acceptance by patients [21].

## Conclusion

In conclusion, the results from our case series showed that PRP is a safe and effective treatment modality for chronic non-healing ulcers. Using PRP to treat chronic wounds/ulcers may not only enhance healing, but also prevent lower extremity amputations caused by non-healing wounds. Therefore, further research and controlled, randomized prospective clinical trials on larger patient population are necessary to validate the results.

## Abbreviations

ACD-A: Anticoagulant citrate dextrose solution - formula A
CaCl_2_: Calcium chloride
FGF: Fibroblast growth factor
IGF: Insulin-like growth factor
PDGF: Platelet derived growth factor
PPP: Platelet poor plasma
PRP: Platelet rich plasma
RBC: Red blood cell
TGF-β: Transforming growth factor – β
VAC: Vacuum assisted closure
VEGF: Vascular endothelial growth factor
WBC: White blood cell
